# High-energy diet modify rumen microbial composition and microbial energy metabolism pattern in fattening sheep

**DOI:** 10.1186/s12917-023-03592-6

**Published:** 2023-02-02

**Authors:** Ting Ge, Chen Yang, Bo Li, Xiaoyu Huang, Leiyun Zhao, Xiaoqiang Zhang, Lintao Tian, Enping Zhang

**Affiliations:** College of Animal Science and Technology, Northwest Agriculture & Forestry University, Xianyang, 712100 Shaanxi China

**Keywords:** Fattening sheep, Dietary energy, Ruminal fermentation, Ruminal microbiota

## Abstract

Higher dietary energy is often used to achieve better animal performance in mutton sheep production. Notably, changing the diet formula affects rumen fermentation and the microbiota of ruminants. In this study, we investigated the effect of dietary energy on rumen fermentation and ruminal microbiota in fattening sheep. Fifteen 2-month-old white-headed Suffolk sheep (♂) × Hu sheep (♀) crossbred lambs were randomly divided into three treatments based on the dietary energy of the feeds fed: 8.67 MJ/kg (Low energy (LE); *n* = 5), 10.38 MJ/kg (standard energy (CON); *n* = 5), and 12.31 MJ/kg (high energy (HE); *n* = 5) groups. After 70 days of feeding, sheep were slaughtered and the ruminal fluids were collected and analyzed to determine fermentation parameters. Microbiota was determined using metagenomics sequencing. Notably, the microbial cell protein (MCP) and butyric acid concentrations were significantly high in the HE group. Metagenomic sequencing revealed that ACE and Chao indexes of the HE group were significantly decreased. Four genera among the major classified taxa across all the kingdoms differed in relative abundance in the three dietary energy levels. The relative abundances of *Prevotella_brevis, Succiniclasticum_ruminis, Prevotellace-ae_bacterium,* and *Lachnospiraceae_bacterium* were significantly correlated with rumen fermentation. Kyoto Encyclopedia of Genes and Genomes (KEGG) pathway enrichment analysis further revealed that a high-energy diet increased lipid metabolism of microbiota. The Carbohydrate Active enzymes (CAZy) gene, which participates in energy metabolism, was upregulated, while genes regulating plant cell wall degradation were downregulated in the HE group. These results suggest that a high-energy diet had minimal influence on the rumen fermentation pattern but altered the composition of the rumen microbiota, enhancing microbial lipid metabolism and limiting crude fiber metabolism. The findings of this study provide scientific evidence of the effect of dietary energy on ruminant fermentation and fattening sheep production.

**Supplementary Information:**

The online version contains supplementary material available at 10.1186/s12917-023-03592-6.

## Introduction

The rumen harbors numerous microorganisms, which form a highly interconnected complex ecosystem [1]. The microbial community comprises bacteria, fungi, protozoa, and archaea, which play distinct and important functions in their respective ecological niches [2]. Ruminants are capable of using crude fibers and converting nonprotein nitrogen into microbial protein because of microbial interactions in the rumen. Ruminants are thus able to utilize energy sources that monogastric animals may not, underlining the indispensable role of microbiota to the host. Studies postulate that ruminal microbes participate in energy and protein metabolisms [3–5], affecting health and host immunity [6–8]. Notably, the nutrient level and rumen ecological niche influence the composition of the rumen microbial community. Therefore, understanding the rumen bacterial community forms a basis for improving ruminant productivity.

In the modern intensive farming mode, concentrated rations are used to increase production by providing high energy to the animal. An increase in dietary energy levels displayed promising results in the performance of lambs, with average daily weight gains increased with metabolic energy elevating [9]. A high-energy dietary formulation alters the rumen micro-ecosystem accordingly, leading to improvements in performance traits [10]. Feeding fattening Hu sheep a high-concentrate corn-based diet improves the performance and rumen fermentation indexes [11]. There are sophisticated exchanges between ruminants and rumen microorganisms. Dietary energy is a pivotal factor affecting the composition and structure of the rumen microbiota [12]. For instance, a grain-based diet lowers the bacterial diversity in cattle rumen compared to a forage-rich diet [13]. High-energy diets increase the relative abundance of Bacteroidetes and Prevotella but reduce the abundance of Proteobacteria. By affecting the bacterial community structure, it alters the synthesis of main metabolites important to animal health [14]. Despite such important findings, little is known about how a high-energy diet that promotes a weight gain of more than 200 g influences rumen fermentation and microbiota.

In this study, the effects of dietary energy levels on rumen fermentation and microbiota in sheep were assessed across the different fattening stages. The interactions between microbes and sheep energy absorption were also analyzed to understand the role of microorganisms in host energy metabolism. The findings of this study provide solid scientific evidence for the best mutton production practice.

## Materials and methods

### Ethical statement

The protocol for this study was approved by the Animal Care and Use Committee of the Northwest Agriculture & Forestry University, China (Approval No. DK2022060).

### Sheep management

The sheep were reared at Gansu Charming Sheep Breeder Co., Ltd. (Qingyang City, Gansu Province) for 78 days between July 24, 2020, and October 10, 2020, and comprised 8 days of pretest and 70 days of testing. Thirty-six 2-month-old white-headed Suffolk sheep (♂) × Hu sheep (♀) crossbred lambs with an average body weight of 20.58 ± 1.47 kg were selected from 2000 lambs born at the same time (sex ratio of the lambs was 1:1). The lambs were randomly divided into the low energy (LE), standard (CON), and high energy (HE) groups based on diet. Every group had four replicates, each with three sheep. The sheep feeds were prepared based on the mutton sheep feeding standard of the China Agricultural Industry Standard (NY/T816-2004). The weight gain of the 20.0 kg sheep was 200 g/d. The digestible energy (DE) and crude protein in the CON diet were 10.38 MJ/kg and 14.36%, respectively. The LE diet contained an 85% energy level compared to that of the standard group, and its DE was 8.67 MJ/kg. The energy in HE feeds was 15% higher than that of the CON diet and had a DE value of 12.31 MJ/kg. Notably, the dietary protein was equal in all groups. The lambs were adequately fed on a mixed ration of dried pellets twice a day at 08:00 and 17:00 and had enough water. Table 1 highlights the dietary composition and nutritional level of the feeds.

**Table 1:** Dietary composition, nutritional level, and energy of feeds fed to the experimental sheep

Item	LE	CON	HE
Ingredient composition, g/kg DM			
Corn	0.00	350.00	370.50
Soybean meal	91.60	144.60	86.50
Wheat barn	278.40	0.00	166.30
Alfalfa hay	200.00	200.00	250.00
Oat hay	100.00	0.00	0.00
Corn stalk	300.00	275.40	66.70
Soybean oil	0.00	0.00	30.00
CaHPO_4_	10.00	10.00	10.00
Premix^a^	20.00	20.00	20.00
Chemical composition^b^			
Digestible energy, MJ/kg	8.67	10.38	12.31
Crude protein, g/kg	143.60	143.60	144.00
Ether extract, g/kg	24.00	23.80	28.50
Neutral detergent fiber, g/kg	455.10	310.90	237.00
Acid detergent fiber, g/kg	232.60	163.20	92.20
Ca, g/kg	0.81	0.75	0.75
P, g/kg	0.67	0.52	0.62

^a^The premix comprised 8000 IU VA, 212000 IU VD, 800 IU VE, 5500 mg Fe, 500 mg Cu, 3500 mg Zn, 4000 mg Mn, 60 mg I, 20 mg Se, and 33.5 mg Co per kg of the diet

^b^Chemical composition is calculated

### Sample collection and measurement

#### Rumen fermentation indexes

One sheep was randomly selected from each replicate, and one of the eight remaining sheep in each group was selected, totaling five sheep per group, for slaughter and sampling on the morning of the 70th day of the trial. The sheep were slaughtered after 12 h of fasting. Rumen fluid was collected and immediately filtered through four layers of sterile gauze. The rumen fluid pH was measured using a portable pH probe (RPB10, Shanghai Haiheng Electromechanical Instrument Co., LTD, Shanghai, China). Each rumen fluid sample was divided into four parts: one part was mixed with 0.2 mol/L hydrochloric acids and stored at − 20 °C before determining the ammonia nitrogen content, while the rest were stored at − 80 °C awaiting the microbial cell protein (MCP) test, volatile fatty acid (VFA) test, and metagenomics sequencing.

The concentration of ammonia nitrogen was determined using the indophenol method after thawing and centrifuging the rumen fluid at 11000×g for 10 min at 4 °C [15]. The MCP was determined by thawing the frozen rumen fluid at room temperature and subsequently centrifuging it at 11000×g for 8 min to remove the unwanted debris. The supernatant was then centrifuged for 20 min at 71000×g, followed by adding 100 μL of the supernatant to 5 mL of Coomassie bright blue solution. The microbial protein concentration was finally measured using a Synergy HT multifunctional microplate reader as previously described [16]. VFA concentration was determined using an Agilent 7820A gas chromatograph (Agilent Technologies, USA) as previously described [17]. Briefly, 5 mL of rumen fluid was incubated at − 20 °C, thawed, and centrifuged at 21000×g for 10 min. Thereafter, 400 μL of metaphosphoric acid was added to 2 mL of the sample supernatant in a 4 mL centrifuge tube, mixed thoroughly, and incubated for 3 - 4 h at 5 °C to precipitate the protein. The mixture was then centrifuged at 21000×g for 15 min at 4 °C to separate the protein from impurities. Crobituric acid (200 μL) was then added to 1 mL of the supernatant, followed by sample agitation at 0.5 °C for 0.5 - 1 h and analysis using gas-liquid chromatography. The column, sample, and detector temperatures were 150 °C, 250 °C, and 250 °C, respectively. The total flow rate of high-purity nitrogen was 30 mL/min, while the column, hydrogen, and airflow rates were 1.4 mL/min, 30 mL/min, and 300 mL/min, respectively.

#### Metagenomics of the rumen microorganisms

Total genomic DNA from rumen fluid samples was extracted using the MP-soil E.Z.N.A.® Soil DNA Kit (Omega Bio-Tek, Norcross, GA, U.S.) following the manufacturer’s instructions. The sequencing was performed on an Illumina NovaSeq/Hiseq Xten platform (Illumina Inc., San Diego, CA, USA) at Majorbio Bio-Pharm Technology Co., Ltd. (Shanghai, China). The generated sequence data were deposited in the NCBI Short Read Archive database (Accession Number: PRJNA826547).

After sequence quality control and genome assembly, gene prediction, taxonomy, and functional annotation were conducted. Kyoto Encyclopedia of Genes and Genomes (KEGG) annotation was performed using the Diamond tool [18] (http://www.diamondsearch.org/index.php, version 0.8.35) based on data in the KEGG database (http://www.genome.jp/keeg/, version 94.2). The cutoff e-value was set at 1e-5. Carbohydrate-active enzymes (CAZy) annotation was conducted using hmm can (http://hmmer.janelia.org/search/hmmscan) against the CAZy database (http://www.cazy.org/) with an e-value cutoff of 1e-5. The detailed method is shown in the supplementary material 1.

### Statistical analysis

Fermentation data were tested for normality using the Shapiro-Wilk test. The data was then analyzed using one-way ANOVA with an LSD post-hoc test, performed using SPSS version 24.0. software (SPSS, Chicago, IL, USA). Metagenomics data were presented as box-and-whiskers plots constructed after a two-tailed Wilcoxon rank-sum test. An FDR correction of the *p*-values was conducted to evaluate the taxonomic data using the default stats packages available in R (V3.6.1). The alpha diversity of bacteria was analyzed using the Mothur tool (Version 1.30.2) and visualized using the R Software (Version 2.15.3). ANOSIM of rumen microbial community composition was analyzed using abund_jaccard. Correlation network analysis was performed using Python (Version 3.5) Networkx package. CAZy and KEGG enrichment data were analyzed using the nonparametric Wilcoxon rank-sum test. The significance threshold was set at *p* < 0.05, and the highly significant threshold at *p* < 0.01.

## Results

### Rumen fermentation indexes

The rumen fermentation indexes are outlined in Table 2. There was no significant difference in NH_3_-N concentration and the pH of rumen fluid among the three groups. Notably, MCP concentration in the rumen fluid of the CON and LE groups was highly significantly lower than that in the HE group (*p* < 0.01). However, there was no significant difference in MCP concentration in the rumen fluid between the CON and LE groups.

**Table 2:** Rumen fermentation indexes of sheep fed on different energy diets

Item	LE	CON	HE	*p*-value
Ammonia nitrogen, mmol/L	8.8 ± 0.39	10.09 ± 0.87	8.38 ± 1.23	0.75
pH	5.98 ± 0.05	5.78 ± 0.20	5.70 ± 0.21	0.25
MCP, μg/mL	1149.88 ± 120.94^a^	1224.08 ± 98.95^a^	2777.78 ± 163.53^b^	0.01
Acetic acid, mmol/L	44.54 ± 3.12^b^	35.37 ± 3.88 ^ab^	32.09 ± 3.48^a^	0.02
Propionic acid, mmol/L	9.57 ± 3.12 ^ab^	12.07 ± 3.88 ^b^	7.22 ± 3.48 ^a^	0.01
Isobutyric acid, mmol/L	0.83 ± 0.09 ^b^	0.94 ± 0.09^b^	0.57 ± 0.06^a^	0.01
Butyric acid, mmol/L	6.48 ± 0.39 ^a^	6.75 ± 0.96 ^a^	12.26 ± 1.01^b^	0.01
Isovaleric acid, mmol/L	0.97 ± 0.11 ^b^	1.18 ± 0.08 ^b^	0.58 ± 0.08 ^a^	0.01
Valeric acid, mmol/L	0.47 ± 0.04 ^a^	0.78 ± 0.12 ^b^	0.60 ± 0.08	0.02
Acetic / Propionic ratio	4.70 ± 0.35 ^ab^	3.10 ± 0.37 ^a^	4.82 ± 0.81^b^	0.04
Total VFA, mmol/L	62.83 ± 3.26	57.07 ± 5.47	53.3 ± 3.67	0.75

Different superscript letters denote the significant difference. Same as below

The dietary energy level altered VFA concentration in the rumen fluid. There was no significant difference in acetic acid concentration between the CON and HE groups. However, acetic acid concentration was significantly higher in the LE group than in the HE group (*p* < 0.05). The propionic acid concentration was significantly higher in the CON than in the HE group (*p* < 0.05). However, there was no significant difference in propionic acid concentration between the HE and LE groups. Butyric acid concentration was highly significantly lower in the CON and LE groups than in the HE group (*p* < 0.01). The concentration of valeric acid was significantly higher in the CON group than in the LE group (*p* < 0.05). The concentration of Isobutyric and Isovaleric acids was significantly higher in the CON and LE groups than in the HE group (*p* < 0.01). The Acetic/Propionic ratio was significantly lower in the CON group than in the HE group (*p* < 0.05). Of note, there was no significant difference in the total VFA concentration among the three groups.

### Metagenomic sequence data

A total of 139.7 Gb of Illumina HiSeq metagenomic sequences for 15 rumen fluid samples was generated. An average of 9.07 Gb of data for each sample was obtained after eliminating low-quality reads and host contaminants. Based on the assembled contigs with an N50 contig length of 1174 bp, 2.80 million non-redundant genes with an average ORF length of 629 bp were identified. The metagenomic sequencing data were annotated at different taxon levels (137 Phyla, 226 Classes, 388 Orders, 754 Families, 2888 Genera, and 15,883 Species in CON group;145 Phyla, 234 Classes, 395 Orders, 762 Families, 2926 Genera, and 16,079 Species in LE group; 126 Phyla, 204 Classes, 351 Orders, 681 Families, 2438 Genera, and 11,840 Species in HE group).

**Fig. 1:**
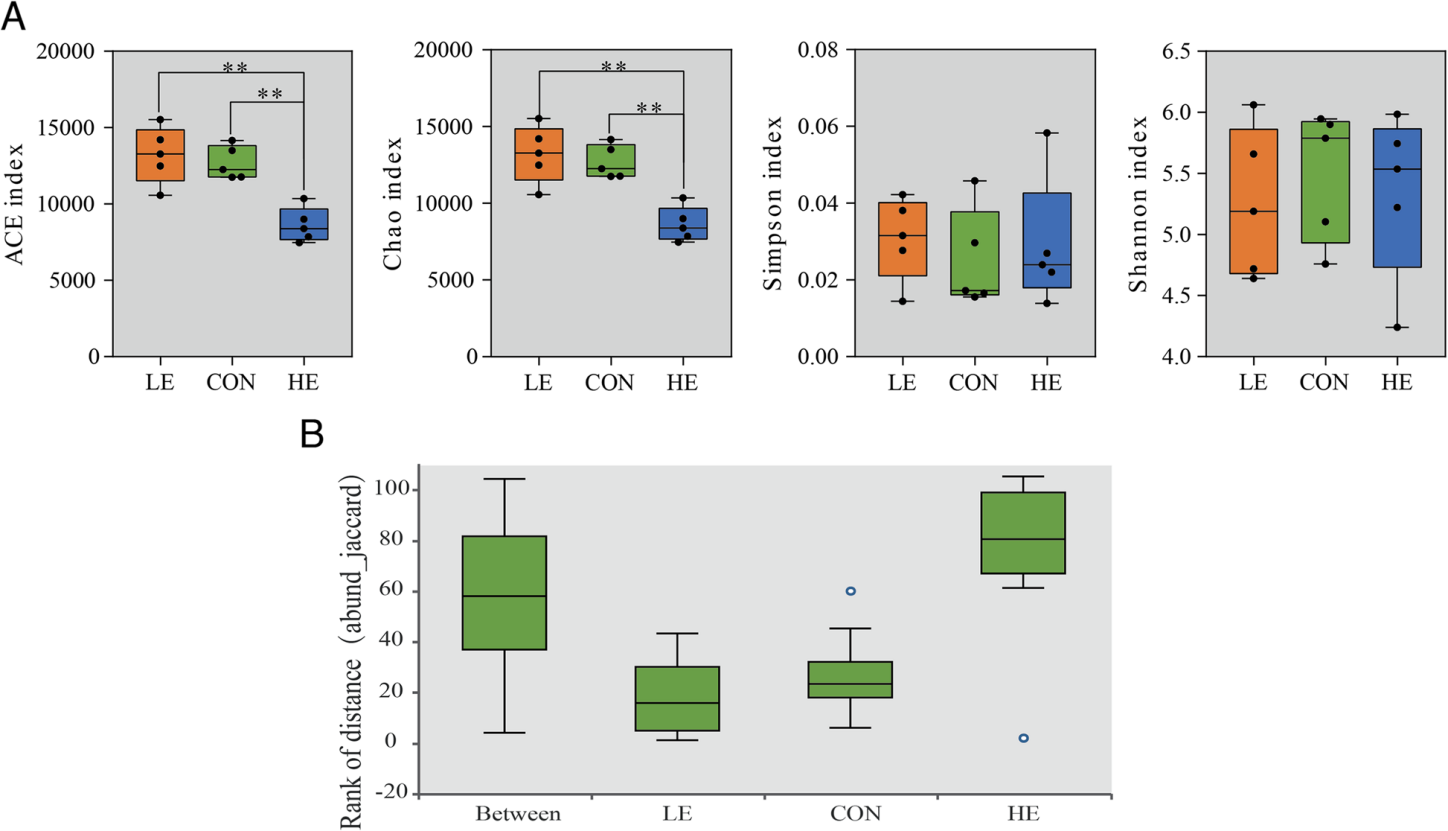
Ruminal microbiota of sheep fed on a diet of different energy levels. **A** Box plots showing the diversity index at the species level. **B** Box plots showing ANOSIM analyses (abund_jaccard) of rumen microbial community composition. *represents *p* < 0.05, ** represents *p* < 0.01

Figure 1 shows the diversity indices of ruminal microflora in sheep fed on diets with different energy levels. The ACE and Chao indexes were significantly higher in the CON and LE groups than in the HE group (*p* < 0.01). ANOSIM analysis revealed a significant difference in the microbial community composition (ANOSIM = 0.328, *p* < 0.01) in the HE group.

### Composition of ruminal microbiota

**Fig. 2:**
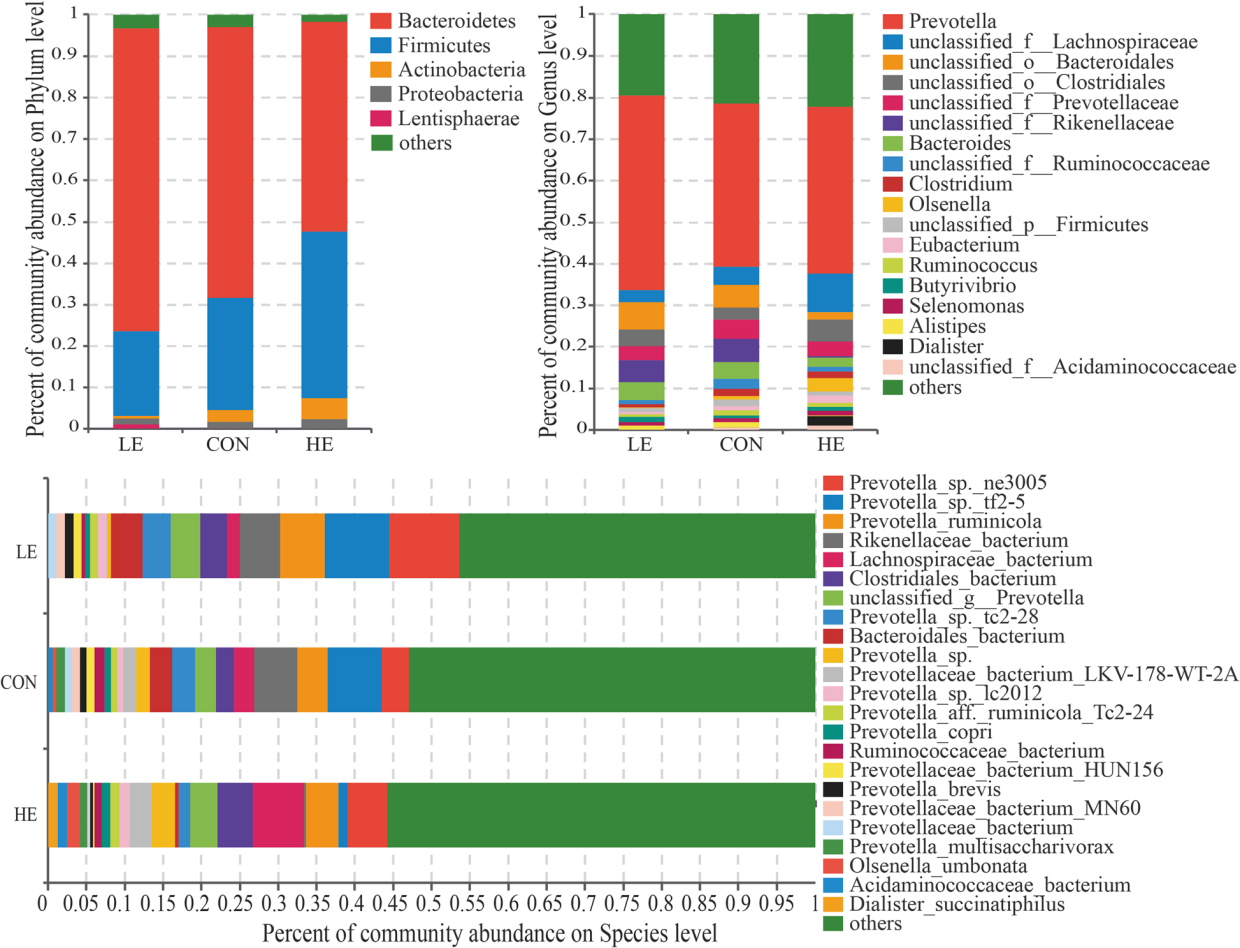
The composition of ruminal microflora in sheep fed on different energy diets. **A** The colored bar plots show the relative abundances of the most dominant phyla (%), genera (%), and species (%)

Different energy diet levels altered the diversity of the ruminal microbiota community in sheep. Bacteroidetes, Firmicutes, Actinobacteria, Proteobacteria, and Lentisphaerae were the dominant phyla in sheep rumen with relative abundances of 65.33, 27.19, 2.74, 1.69, and 0.12% in the CON group, 73.18, 20.40, 0.73, 1.46, 1.03% in the LE group, and 50.74, 40.17, 5.11, 2.37, and 0.02% in the HE group (Fig. 2). The dominant genera across the three energy groups are shown in Fig. 2A. The top 10 most dominant genera in the three energy groups were *Prevotella, unclassified Lachnospiraceae, unclassified Bacteroidales, unclassified Clostridiales, unclassified Prevotellaceae, unclassified Rikenellaceae, Bacteroides, unclassified Ruminococcaceae, Clostridium,* and *Olsenella.* The relative abundance of *unclassified Lachnospiraceae* was significantly lower (*p* < 0.05) in the LE group than in the HE group, while those of *unclassified Bacteroidales* and *Bacteroides* in the CON and LE groups were significantly higher than in the HE group (*p* < 0.01, *p* < 0.05). The relative abundance of *unclassified Rikenellaceae* was significantly higher in the CON group than in the HE group (*p* < 0.05) (Table 3). The top 10 most dominant species across the three energy groups were *Prevotella ne3005, Prevotella tf2-5, Prevotella_ruminicola, Rikenellaceae_bacterium, Lachnospiraceae_bacterium, Clostridiales_bacterium, unclassified Prevotella, Prevotella tc2-28, Bacteroidales_bacterium,* and *Prevotella_sp.* (Fig. 2). The relative abundance of *Rikenellaceae_bacterium* was significantly higher in the CON group than in the HE group (*p* < 0.05), while that of *Lachnospiraceae_bacterium* was significantly higher in the HE group than in the LE group (*p* < 0.05). Moreover, the relative abundance of *Bacteroidales_bacterium* was significantly lower in the HE group than in the CON (*p* < 0.05) and LE (*p* < 0.01) groups (Table 4).

**Table 3:** Top 10 dominant genera of rumen microbiota with different energy intake (%)

Item	LE	CON	HE	*p*-value
*Prevotella*	46.47 ± 6.84	38.25 ± 6.87	36.08 ± 12.73	0.44
*unclassified_Lachnospiraceae*	3.02 ± 0.99^a^	4.30 ± 1.85^ab^	9.39 ± 2.56^b^	0.03
*unclassified_Bacteroidales*	6.32 ± 0.89^b^	5.54 ± 1.61^b^	1.65 ± 0.34^a^	0.01
*unclassified_Clostridiales*	4.11 ± 1.56	3.10 ± 0.94	7.13 ± 3.39	0.22
*unclassified_Prevotellaceae*	3.31 ± 0.59	4.48 ± 1.39	2.90 ± 1.78	0.42
*unclassified_Rikenellaceae*	4.81 ± 1.75^ab^	5.88 ± 2.59^b^	0.29 ± 0.06^a^	0.04
*Bacteroides*	4.31 ± 0.40^b^	4.09 ± 0.66^b^	2.27 ± 0.42a	0.01
*unclassified_Ruminococcaceae*	0.91 ± 0.25	2.56 ± 0.72	1.53 ± 0.68	0.06
*Clostridium*	0.99 ± 0.35	1.77 ± 0.58	1.76 ± 0.47	0.26
*Olsenella*	0.10 ± 0.05	0.83 ± 0.38	3.02 ± 1.86	0.08

**Table 4:** Top 10 dominant species of rumen microbiota with different energy intake (%)

Item	LE	CON	HE	*p*-value
*Prevotella ne3005*	8.39 ± 1.95	3.51 ± 1.23	4.88 ± 3.30	0.31
*Prevotella tf2-5*	9.48 ± 4.00	6.9 ± 3.34	1.25 ± 0.55	0.07
*Prevotella_ruminicola*	5.54 ± 1.12	3.88 ± 1.30	4.05 ± 2.58	0.52
*Rikenellaceae_bacterium*	4.81 ± 1.75^ab^	5.88 ± 2.59^b^	0.29 ± 0.06^a^	0.04
*Lachnospiraceae_bacterium*	1.80 ± 0.61^a^	2.60 ± 1.21^ab^	6.53 ± 1.82^b^	0.02
*Clostridiales_bacterium*	3.58 ± 1.43	2.53 ± 0.84	6.43 ± 3.30	0.21
*unclassified Prevotella*	3.63 ± 0.74	2.70 ± 0.64	3.26 ± 1.77	0.58
*Prevotella tc2-28*	3.60 ± 0.94	2.83 ± 1.44	1.53 ± 0.75	0.40
*Bacteroidales_bacterium*	4.00 ± 0.77^b^	3.00 ± 1.04^b^	0.57 ± 0.13^a^	0.01
*Prevotella_sp.*	0.45 ± 0.04	1.67 ± 1.01	2.40 ± 1.88	0.28

### Correlation network between microbial and ruminal fermentation indexes

**Fig. 3:**
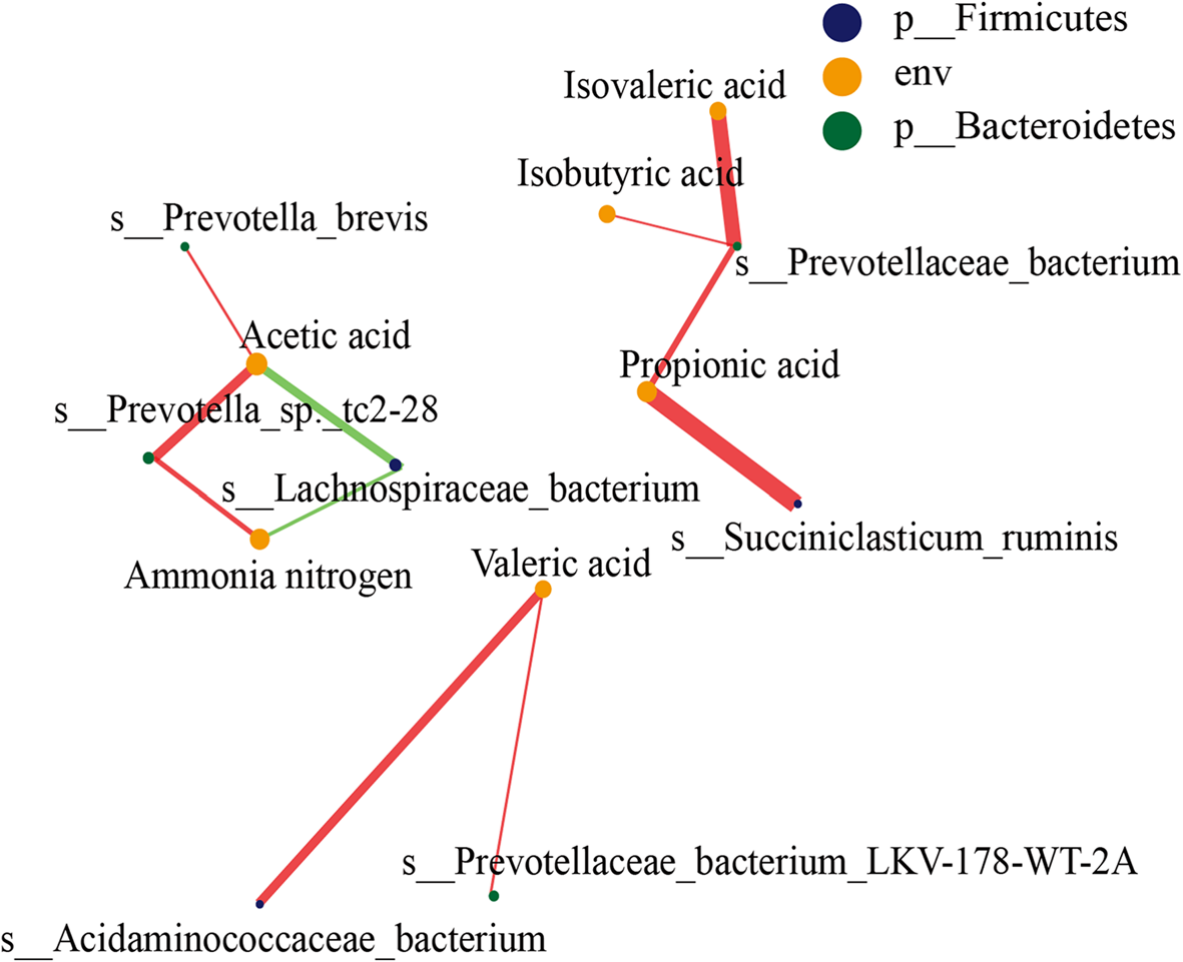
The correlation network between rumen microflora and diet energy level. The correlation network analysis between microbial species abundance and rumen fermentation indexes. Spearman’s rank correlation coefficient > 0.60; *p* < 0.05. Different colors represent different phyla in the rumen. The size of the nodes is proportional to the relative abundance of a species; the red lines indicate a positive correlation between species, while the green lines indicate a negative correlation between species. The thickness of the lines indicate the strength of correlation

The relationship between rumen microbiota and fermentation was analyzed based on the Spearman correlation coefficient. Only ammonia nitrogen, acetic acid, propionic acid, isobutyric acid, isovaleric acid, and valeric acid levels were associated with the abundance of the rumen microbes (Fig. 3). Ammonia nitrogen levels were positively correlated with *Prevotella_sp._tc2-28* abundance, but negatively correlated with *Lachnospiraceae_bacterium* abundance. Acetic acid level was positively correlated with *Prevotella_sp._tc2-28* and *Prevotella_brevis* abundance but negatively correlated with *Lachnospiraceae_bacterium* abundance. Propionic acid level was positively correlated with *Prevotellaceae_bacterium* and *Succiniclasticum_ruminis* abundance. Similarly, isobutyric and isovaleric acid contents were positively correlated with *Prevotellaceae_bacterium* abundance. Valeric acid level was positively correlated with *Prevotellaceae_bacterium_LKV-178* and *Acidaminococcaceae_bacterium* abundance. The relative abundance of rumen microbiota was further calculated to explore variations in the microbial correlation network (Table 5). The relative abundance of *Lachnospiraceae_bacterium* was significantly lower in the LE group than in the HE group (*p* < 0.05). However, the relative abundance of *Prevotella_brevis, Prevotellaceae_bacterium,* and *Succiniclasticum_ruminis* were significantly higher in the CON and LE groups than in the HE group (*p* < 0.05).

**Table 5:** The relative abundance of correlation network related rumen microorganism (%)

Item	LE	CON	HE	*p*-value
*Lachnospiraceae_bacterium*	1.80 ± 0.61^a^	2.60 ± 1.21^ab^	6.53 ± 1.82^b^	0.02
*Prevotella tc2-28*	3.60 ± 0.94	2.83 ± 1.44	1.53 ± 0.75	0.20
*Prevotellaceae_bacterium_LKV-178-WT-2A*	0.10 ± 0.02	1.57 ± 1.20	2.20 ± 1.86	0.26
*Prevotella_brevis*	1.18 ± 0.17^b^	0.90 ± 0.23^b^	0.32 ± 0.16^a^	0.01
*Prevotellaceae_bacterium*	1.05 ± 0.15^b^	0.9 ± 0.18^b^	0.24 ± 0.05^a^	0.01
*Acidaminococcaceae_bacterium*	0.06 ± 0.01	0.57 ± 0.29	0.89 ± 0.54	0.11
*Succiniclasticum_ruminis*	0.67 ± 0.12^b^	0.8 ± 0.13^b^	0.15 ± 0.06^a^	0.01

### Rumen microbial function

**Fig. 4:**
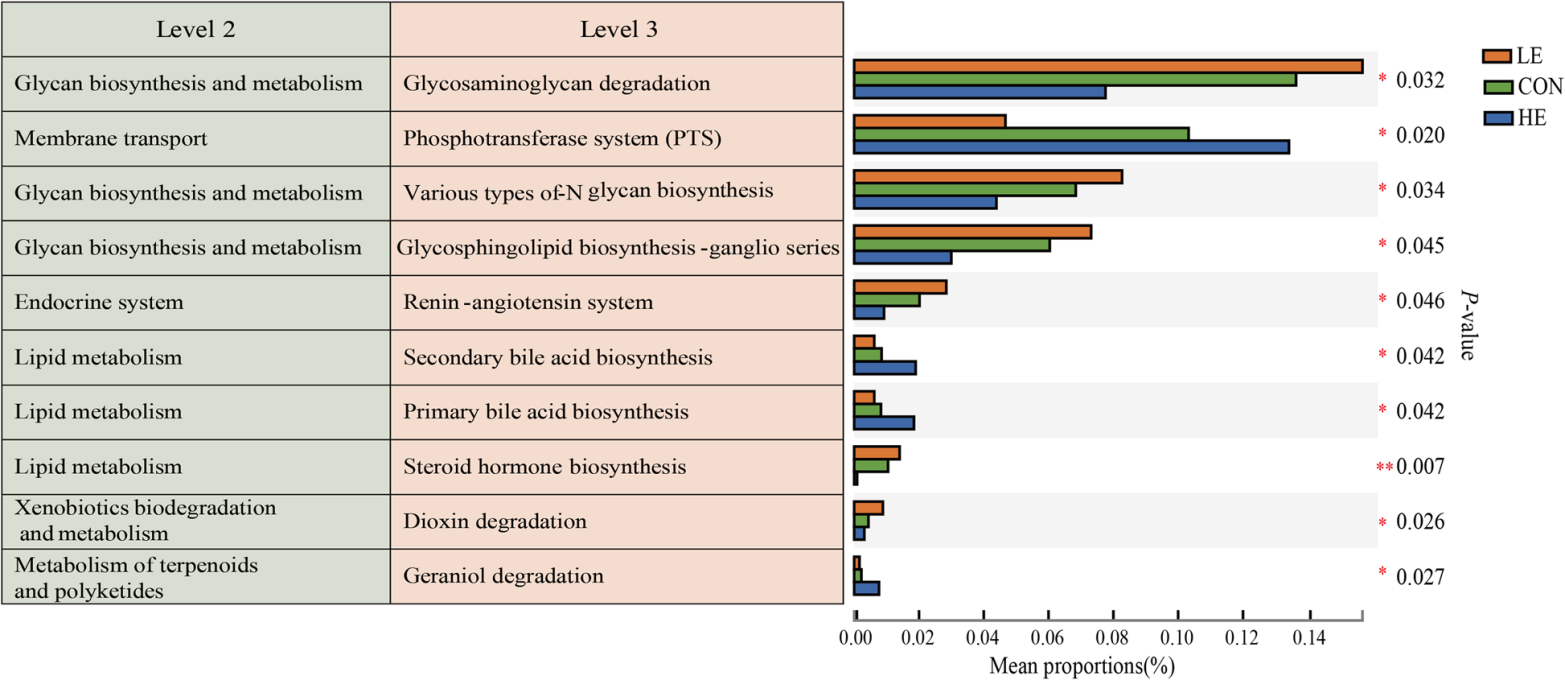
Multi-group comparison of the functional composition of the rumen microbiota in sheep fed on different energy diets

KEGG pathway analysis was performed to investigate the function of the different rumen microbiota. Figure 4 shows the KEGG level 2 functions of the rumen microbiota. KEGG level 2 pathway analysis revealed a significant difference in glycan biosynthesis and metabolism, membrane transport, endocrine system, lipid metabolism, xenobiotics biodegradation and metabolism, metabolism of terpenoids, and polyketides across the three energy groups. KEGG level 3 pathway enrichment analysis revealed that glycosaminoglycan degradation, biosynthesis of numerous N-glycans, glycosphingolipid biosynthesis-ganglio series, and renin and steroid hormone biosynthesis significantly decreased (*p* < 0.05) with an increase in diet energy level. In contrast, the phosphotransferase system (PTS), primary bile acid biosynthesis, secondary bile acid biosynthesis, and geraniol degradation were significantly higher in the HE group (*p* < 0.05) (Fig. 4).

**Fig. 5:**
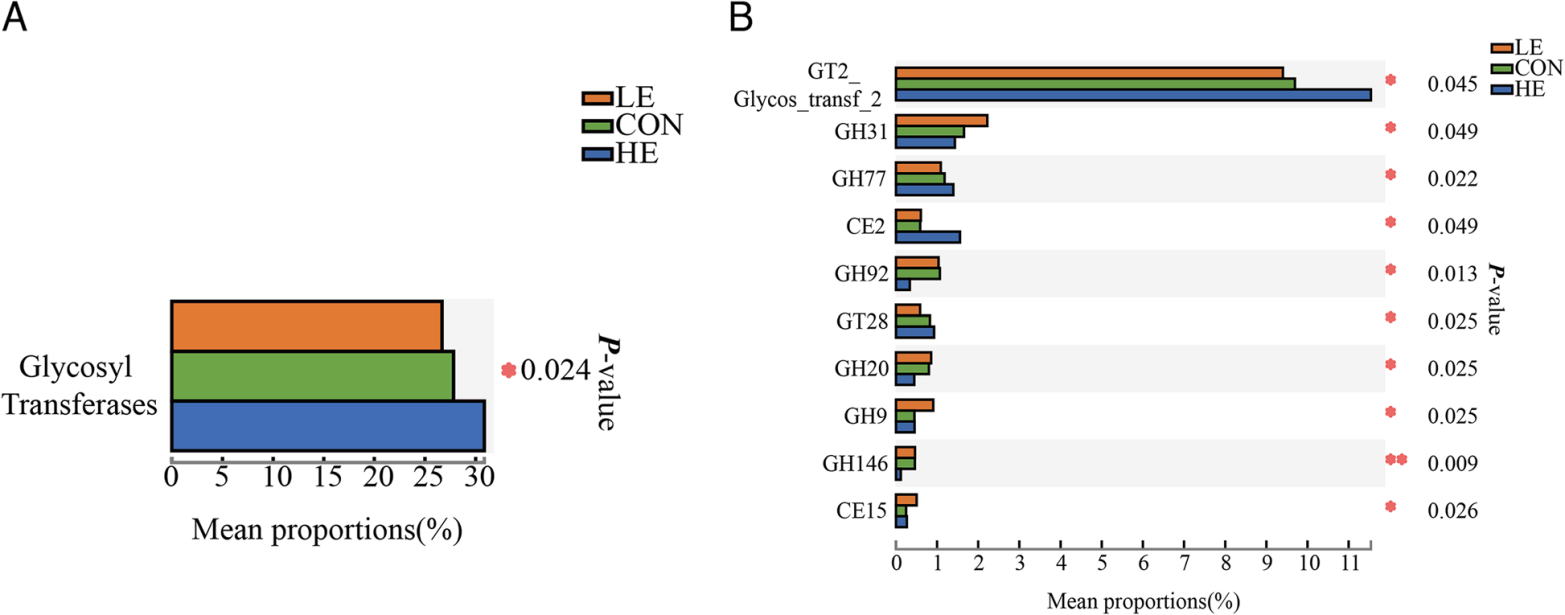
Multi-group comparison of the expression of the CAZy gene in the rumen microbiota of sheep fed on different energy diets. **A** The variation in the expression of the CAZy gene at different dietary energy diets at the class level. **B** The variation in the expression of the CAZy gene at different dietary energy iets at the family level. GTs: Glycosyl Transferases; GHs: Glycoside Hydrolases; CEs: Carbohydrate Esterases

The expression of the CAZy gene encoding carbohydrate catabolism enzymes in the sheep rumen microbiota was analyzed using the nonparametric Wilcoxon rank-sum test. At the class level, the relative expression of the GTs gene was significantly high in the HE group (*p* < 0.05) (Fig. 5 A). At the family level, the relative abundance of GT2_Glycos_transf_2, GH77, CE2, and GT28 genes increased with the dietary energy level (*p* < 0.05). These genes encoded energy metabolism genes, such as 4-α-glucanotransferase (EC 2.4.1.25), acetyl xylan esterase (EC 3.1.1.72), 1,2-diacylglycerol, and 3-β-glucosyltransferase (EC 2.4.1.157). The expression of GH31, GH92, GH9, GH146, and CE15 decreased with an increase in the dietary energy level (*p* < 0.05) (Fig. 5 B). These genes encoded plant cell wall degradation enzymes, such as α-glucosidase (EC 3.2.1.20), α-1,2-mannosidase (EC 3.2.1.-), and endoglucanase (EC 3.2.1.4), β-L-arabinofuranosidase (EC 3.2.1.185), and 4-O-methyl-glucuronoyl methylesterase (EC 3.1.1.-).

## Discussion

The rumen is the most efficient bioreactor, an inseparable relationship exists between rumen microbes and the host. The rumen microbiota comprises bacteria, protozoa, fungi, and archaea. These microorganisms exist in symbiotic, competitive, predation, or antagonistic relationships and are jointly responsible for microbial fermentation in the rumen [19]. Research on rumen microbiota and the host mainly focuses on how nutrition strategies influence the rumen microbiome and how the alteration of the rumen microbiota affects the host.

Dietary energy is an important factor that affects nutrient intake, digestion, metabolic efficiency, and production performance. Herein, high dietary energy levels increased the final weight, live weight, average daily gain, and feed rewards in lambs. The increase of dietary energy significantly increased (*p* < 0.05) the mRNA expression of fat deposition-related genes in subcutaneous fat (HSL), tail fat (*FASN*), and longissimus dorsi (*FABP4*) [20]. The influence of dietary energy on rumen fermentation and microbiota was further investigated because of its crucial role in nutrient digestion and utilization in ruminants.

### High-energy diet affects ruminal fermentation

VFA, NH_3_-N, and MCP are the critical indices of the rumen fermentation pattern. In ruminants, feeds first undergo microbial fermentation in the rumen. Ruminal microbiota breaks down carbohydrates into VFA, the main metabolite of microbial fermentation [21]. Microbes first break down carbohydrates (crude fiber, starch, and soluble sugar) to pyruvate. The pyruvate is then metabolized to different VFAs (acetic acid, propionic acid, butyric acid, isobutyric acid, valeric acid, and isovaleric acid) through different metabolic pathways. Proteins and fats can also be degraded to VFA (mostly brunch chain fatty acids). Ruminants, therefore, utilize VFA, which is degraded from carbohydrates by ruminal microbes rather than glucose. The VFA accounts for 70% ~ 80% metabolizable energy (ME) of ruminant energy needs [22]. ATP for microbial growth is thus released during the VFA synthesis processes.

Rumen fermentation patterns usually change under different nutritional conditions. VFA concentration increases with an increase in the energy level or a high concentrate diet. High ME also significantly increases the proportions of butyrate and valerate [10]. Moreover, high acetate and butyrate concentrations are associated with the intake of high grain diet [23]. In this study, butyric acid levels in the rumen fluid increased with diet energy concentration. A significant increase in MCP with energy intake was also observed, consistent with previous findings [24]. The feed energy level is directly proportional to the rumen MCP yield. However, there was no positive correlation between acetic acid, propionic acid, and daily feed energy in this study, possibly because of a strong correlation between rumen VFA concentration, feed type, and nutrient level [25]. The LE diet contained more crude fibers as the substrate for microbial fermentation to produce VFA. Notably, a high-energy diet had limited influences on the rumen fermentation pattern and provided sufficient energy for microorganisms to synthesize MCP, giving sheep more rumen-protected proteins to metabolize.

### High-energy diet changes the composition of ruminal microbiota

Bacteria are the most abundant and diverse microbiota in the rumen, a phenomenon attributable to diet [26]. The microbiota also affects animal health. Therefore, understanding the effect of dietary energy on rumen microflora is of great significance to animal husbandry. A more diverse microbial community increases the resilience, resistance, and stability of the rumen ecosystem [27]. Diet energy affects the overall rumen multi-kingdom microbiota [28]. Generally, ruminal microbiota diversity decreases with dietary energy [29, 30]. Herein, ACE and Chao indices were significantly higher in the LE group than in the CON and HE groups, consistent with previous studies [31, 32], indicating that a high-energy diet reduces the richness of the microbiome.

Despite the complexity of the rumen ecosystem, the composition and abundance of rumen microbiota are relatively stable at the phylum level. Firmicutes, Bacteroidetes, Fibrobacteres, and Proteobacteria are the four most abundant phyla in the sheep rumen, whereas the dominant genera include *unidentified Prevotellaceae*, *Fibrobacter*, *unidentified Lachnospiraceae*, *Saccharofermentans*, and *Succinivibrio* [31]. In this study, the rumen microbiota was similar with former study despite the wide variation in diet formulation. The dominant rumen microbial phyla were Bacteroidetes, Firmicutes, Actinobacteria, and Proteobacteria whereas the dominant genera were *Lentisphaera*, *Prevotella*, *unclassified Lachnospiraceae, unclassified Bacteroidales, unclassified Clostridiales,* and *unclassified Prevotellaceae*.

Relevant studies continue to reveal the functions of ruminal microbiota in ruminants. For instance, Bacteroidetes degrade carbohydrates and polysaccharides more efficiently than Proteobacteria [33]. At the genus level, *Butyrivibrio*, *Fibrobacter*, *Olsenella,* and *Prevotella* are critical in degrading cellulose [34]. Generally, studies postulate that a high-energy diet reduces the relative abundance of ruminal microbiota that participate in crude fiber fermentation [35–37]. A previous study revealed that *Bacteroidetes* utilize crude fiber in the form of glycans (oligomeric and polymeric glycans); the abundance of this bacteria is influenced by the dietary intake of these indigestible carbohydrates [38]. In this study, the relative abundance of *Bacteroides* in the HE group was relatively low (feed of sheep in the HE group contained little crude fiber). Furthermore, the relative abundance of *Rikenellaceae_bacterium* and *Bacteroidales_bacterium* decreased with an increase in the dietary energy level. *Rikenellaceae* is one of the main producers of VFA. Previous studies postulate that a high-fat diet significantly decreases the *Rikenellaceae* composition in the rumen [39]. Similarly, there was a decrease in the relative abundance of crude fiber metabolizing bacteria in the rumen with an increase in energy diet.

The rumen microbiota produces numerous metabolites, including VFA and polyamines, through anaerobic fermentation. Notably, 10% of metabolites in mammalian blood are derived from microbes or microbial activities [40]. The blood metabolites participate in developing and regulating host physiology and immunity and can be modified into other novel metabolites [41]. The relationships between some microorganisms and rumen metabolites have been well established. For instance, members of the *Lachnospiraceae* family are among the main producers of short-chain fatty acids [42]. *Bacteroidetes* produce enzymes that degrade plant cell wall compounds (e.g., cellulose and pectin) to release VFA (mainly acetate, propionate, and butyrate) [43]. The phylum Lentisphaerae is associated with changes in feed efficiency [44]. *Succiniclasticum* degrades starch into acetic and succinic acids and further converts succinic acid into propionic acid [45]. The abundance of *Prevotellaceae UCG-003* is positively correlated with the rumen VFA content [46]. In this study, correlation analysis revealed that only the relative abundance of *Lachnospiraceae_bacterium, Prevotella_brevis, Prevotellaceae_bacterium, and Succiniclasticum_ruminis* was significantly different among the groups. *Prevotella_brevis* promotes acetic acid synthesis, *Succiniclasticum_ruminis* promotes synthesis, *Prevotellaceae_bacterium* promotes propionic acid, isobutyric acid, and isovaleric acid synthesis, whereas *Lachnospiraceae_bacterium* inhibits ammonia nitrogen and propionic acid synthesis.

### High-energy diet alters rumen microbial function

Changes in the KEGG pathway reflect an alteration in the rumen microbial function under specific conditions. Changes in enzyme function also modify microbial function. Rumen microbial functions changes with dietary energy concentration. Zhang et al. [47] investigated the effect of feeding sheep with caragana, corn straw, and alfalfa. Sheep fed with caragana had higher DE and mainly enhanced the microbial function on starch and sucrose metabolism, fructose and mannose metabolism, photosynthesis, and D-alanine metabolism in the rumen. In a separate study, Wang et al. [10] confirmed that a higher energy digestible diet increased the abundance of bacteria associated with carbohydrate metabolism. In this study, the expression of genes associated with lipid metabolism increased with dietary energy. However, a reverse trend was observed for genes associated with glycan biosynthesis and metabolism. Microbial CAZy regulates carbohydrate metabolism and is thus critical to the energy available to the host. Herein, a high-energy diet increased the expression of genes involved in energy metabolism. However, the expression of genes that encode enzymes that catalyze plant cell wall degradation was decreased.

KEGG and CAZy enrichment data further revealed that a high-energy diet improved lipid metabolism in sheep by promoting the expression of genes associated with lipid metabolism. In contrast, low-energy diets enhanced glycan biosynthesis and metabolism by promoting the expression of enzymes involved in plant cell wall degradation to meet the animals’ energy needs.

Although we observed improvements in production performance caused by higher dietary energy in our previous study and obtained microbial-related data in this current study, we could not directly correlate microbial-related data with host metabolism. This limitation was occasioned by preliminary sample collection issues, which made it difficult to facilitate such correlations. Future studies should focus on this aspect to have holistic results.

## Conclusion

A high dietary energy diet had limited influence on the rumen fermentation pattern but increased MCP synthesis. Notably, changes in dietary energy altered the rumen microbial composition and microbial energy metabolism pattern. In particular, a high dietary energy diet reduced crude fiber metabolism but strengthened the energy metabolism pathway of microorganisms. This study recommends a fattening dietary energy level of 12.31 MJ/kg for 2-month-old lambs.

## Supplementary Information

**Additional file 1: **Metagenomics of the rumen microorganisms.

## Data Availability

The datasets generated and/or analyzed are available in the NCBI Short Read Archive database repository [https://www.ncbi.nlm.nih.gov/bioproject/PRJNA826547, Accession Number: PRJNA826547].

## Abbreviations

ANOSIM: Analysis of similarity
CAZy: Carbohydrate-active enzymes
CEs: Carbohydrate esterases
GTs: Glycosyl transferases
GHs: Glycoside hydrolases
MCP: Microbial cell protein
KEGG: Kyoto encyclopedia of genes and genomes
VFA: Volatile fatty acid
