# Supplementary material for: High-energy diet modify rumen microbial composition and microbial energy metabolism pattern in fattening sheep
Source: BMC Vet Res. 2023 Feb 2;19:32. doi: 10.1186/s12917-023-03592-6 (PMC9893671; doi:10.1186/s12917-023-03592-6)
Supplement: Supplementary file 1 — Additional file 1: Metagenomics of the rumen microorganisms. [file 12917_2023_3592_MOESM1_ESM.docx]

**Metagenomics of the rumen microorganisms**

Total genomic DNA from rumen fluid samples was extracted using the MP-soil E.Z.N.A.® Soil DNA Kit (Omega Bio-Tek, Norcross, GA, U.S.) following the manufacturer's instructions. DNA concentration and purity were determined using TBS-380 and NanoDrop2000, respectively, while the quality was checked on a 1% agarose gel.

The DNA was fragmented into about 400 bp segments using Covaris M220 (Gene Company Limited, China) for paired-end library construction done using the NEXTflexTM Rapid DNA-Seq platform (Bioo Scientific, Austin, TX, USA). Adapters containing the full sequences complementary to the sites of interest were ligated to the blunt end of the DNA fragments. Paired-end sequencing was performed on an Illumina NovaSeq/Hiseq Xten platform (Illumina Inc., San Diego, CA, USA) at Majorbio Bio-Pharm Technology Co., Ltd. (Shanghai, China) using NovaSeq Reagent Kits/HiSeq X Reagent Kits, following the manufacturer's instructions (www.illumina.com). The generated sequence data were deposited in the NCBI Short Read Archive database (Accession Number: PRJNA826547).

The raw reads were trimmed and cleaned by removing adaptor sequences and low-quality reads (reads with N bases, a minimum length threshold of 50 bp, and a minimum quality threshold of 20) using the fast tool (Chen et al., 2018) (https://github.com/OpenGene/fastp, version 0.20.0) on the Majorbio Cloud Platform (cloud.majorbio.com). Clean reads were mapped to the Ovis aries (assembly Oar_rambouillet_v1.0) reference genome using BWA (Li and Durbin, 2009) (https://www.ncbi.nlm.nih.gov/genome/?term=SHEEP) tools to identify and remove reads originating from the host (sheep). The high-quality reads were then assembled into contigs using MEGAHIT (Li et al., 2015) (Parameters: kmer_min=47，kmer_max=97，step=10) (https://github.com/voutcn/megahit, version 1.1.2), which employs succinct de Bruijn graphs. Contigs larger than 300 bp were selected for final assembly.

Open reading frames (ORFs) in the contigs were identified using the MetaGene tool as previously described by Noguchi et al. (2006) (http://metagene.cb.k.u-tokyo.ac.jp/). The predicted ORFs larger than 100 bp were translated into amino acid sequences using the NCBI translation table (http://www.ncbi.nlm.nih.gov/Taxonomy/taxonomyhome.html/index.cgi?chapter=tgencodes#SG1).

A non-redundant gene catalog with 90% sequence identity and 90% coverage was constructed using the CD-HIT online tool (http://www.bioinformatics.org/cd-hit/, version 4.6.1) as described by Fu et al. (2012). Quality reads with 95% identity were mapped to the non-redundant genes using the SOAPaligner online tool (Li et al., 2008) (http://soap.genomics.org.cn/, version 2.21). The gene abundance in each sample was then evaluated.

Annotation of the non-redundant genes based on the NCBI NR database was performed using the blast tool in the DIAMOND software, v 0.9.19. The cutoff e-value for taxonomic annotations was set at 1e-5 using the Diamond online tool (Buchfink et al., 2015) (http://www.diamondsearch.org/index.php, version 0.8.35). A cluster of orthologous groups of proteins (COG) annotation for selected sequences was performed using the same tool (Buchfink et al., 2015) (http://www.diamondsearch.org/index.php, version 0.8.35) based on data in the eggNOG database (version 4.5.1). The cutoff e-value was set at 1e-5. Differences in microbiota composition among groups were assessed using Analysis of similarity (ANOSIM) of (abund_jaccard) distances. Kyoto Encyclopedia of Genes and Genomes (KEGG) annotation was performed using the Diamond tool (Buchfink et al., 2015) (http://www.diamondsearch.org/index.php, version 0.8.35) based on data in the KEGG database (http://www.genome.jp/keeg/, version 94.2), with the cutoff e-value set at 1e-5. Carbohydrate-active enzymes (CAZy) annotation was conducted using hmm can (http://hmmer.janelia.org/search/hmmscan) against the CAZy database (http://www.cazy.org/) with an e-value cutoff of 1e-5.

### References

Buchfink, B., Xie, C., Huson, D.H., 2015. Fast and sensitive protein alignment using DIAMOND. Nature methods 12, 59-60. DOI: 10.1038/nmeth.3176.

Chen, S., Zhou, Y., Chen, Y., Gu, J., 2018. fastp: an ultra-fast all-in-one FASTQ preprocessor. Bioinformatics (Oxford, England) 34, i884-i890. DOI: 10.1093/bioinformatics/bty560.

Fu, L., Niu, B., Zhu, Z., Wu, S., Li, W., 2012. CD-HIT: accelerated for clustering the next-generation sequencing data. Bioinformatics (Oxford, England) 28, 3150-3152. DOI: 10.1093/bioinformatics/bts565.

Li, D., Liu, C.M., Luo, R., Sadakane, K., Lam, T.W., 2015. MEGAHIT: an ultra-fast single-node solution for large and complex metagenomics assembly via succinct de Bruijn graph. Bioinformatics (Oxford, England) 31, 1674-1676. DOI: 10.1093/bioinformatics/btv033.

Li, H., Durbin, R., 2009. Fast and accurate short read alignment with Burrows-Wheeler transform. Bioinformatics (Oxford, England) 25, 1754-1760. DOI: 10.1093/bioinformatics/btp324.

Li, R., Li, Y., Kristiansen, K., Wang, J., 2008. SOAP: short oligonucleotide alignment program. Bioinformatics (Oxford, England) 24, 713-714. DOI: 10.1093/bioinformatics/btn025.

Noguchi, H., Park, J., Takagi, T., 2006. MetaGene: prokaryotic gene finding from environmental genome shotgun sequences. Nucleic acids research 34, 5623-5630.
